# Real-World Effectiveness of Palbociclib Plus Aromatase Inhibitors in African American Patients With Metastatic Breast Cancer

**DOI:** 10.1093/oncolo/oyad209

**Published:** 2023-07-24

**Authors:** Hope S Rugo, Xianchen Liu, Benjamin Li, Lynn McRoy, Connie Chen, Rachel M Layman, Adam Brufsky

**Affiliations:** Department of Medicine, University of California San Francisco Helen Diller Family Comprehensive Cancer Center, San Francisco, CA, USA; Pfizer Inc., New York, NY, USA; Pfizer Inc., New York, NY, USA; Pfizer Inc., New York, NY, USA; Pfizer Inc., New York, NY, USA; Department of Breast Medical Oncology, Division of Cancer Medicine, The University of Texas MD Anderson Cancer Center, Houston, TX, USA; Department of Medicine, Division of Hematology/Oncology, UPMC Hillman Cancer Center, University of Pittsburgh Medical Center, Pittsburgh, PA, USA

**Keywords:** breast cancer, African American, aromatase inhibitors, cyclin-dependent kinases

## Abstract

**Background:**

Disparities in survival and clinical outcomes between African American and White patients with breast cancer (BC) are well documented, but African American patients have not been well represented in randomized clinical trials of CDK4/6 inhibitors. Real-world studies can provide evidence for effective treatment strategies for underreported patient populations.

**Patients and Methods:**

This retrospective analysis of African American patients with HR+/HER2− metastatic breast cancer (mBC) from the Flatiron Health longitudinal database evaluated treatments for patients with BC in routine clinical practice in the US. Patients initiated first-line therapy with palbociclib plus an aromatase inhibitor (AI) or AI alone between February 2015 and March 2020. Outcomes assessed included overall survival (OS) and real-world progression-free survival (rwPFS) until September 2020.

**Results:**

Of 270 eligible patients, 127 (median age 64 years) were treated with palbociclib + AI, and 143 (median age 68 years) were treated with an AI. Median follow-up was 24.0 months for palbociclib + AI and 18.2 months for AI-treated patients. Median OS was not reached (NR; 95% CI, 38.2-NR) in the palbociclib + AI group versus 28.2 months (95% CI, 19.2-52.8) in the AI group (adjusted HR, 0.56; 95% CI, 0.36-0.89; *P* = .013). Median rwPFS was 18.0 months (95% CI, 12.4-26.7) in the palbociclib + AI group and 10.5 months (95% CI, 7.0-13.4) in the AI group (adjusted HR, 0.74; 95% CI, 0.47-1.17; *P* = .199).

**Conclusion:**

This comparative analysis of palbociclib + AI versus AI alone indicates that palbociclib combined with endocrine therapy in the first line is associated with improved effectiveness for African American patients with HR+/HER2− mBC in real-world settings.

**Trial number:**

NCT05361655

## Teaser

The results reported in this article provide evidence of the effectiveness of palbociclib in combination with an aromatase inhibitor for first-line treatment of African American patients with HR+/HER2– metastatic breast cancer.

**Implications for Practice**

Results from this study provide additional evidence for the effectiveness of palbociclib in combination with an aromatase inhibitor for first-line treatment of African American patients with HR+/HER2− metastatic breast cancer.

## Introduction

Racial disparities in the cancer burden experienced by patients in the US have been well documented. African Americans in particular have higher mortality rates from cancers than any other broadly defined racial or ethnic group.^1^ Breast cancer (BC) is the leading cause of cancer death for African American women, and it is estimated that 6800 African American women would die from this disease in 2022.^2^ Although BC incidence is lower in African American women compared with White women, the mortality rate is 41% higher, according to 2015-2019 data.^2^ A recent study has found that these survival outcome disparities exist across BC molecular subtypes, including hormone receptor-positive/human epidermal growth factor receptor 2-negative (HR+/HER2−) BC.^3^ The HR+/HER2− subtype accounts for the majority (68%) of BC cases.^4^

As of 2018, it is estimated that over 140 000 patients are living with metastatic breast cancer (mBC) in the US, and this number is predicted to increase to over 169 000 by 2025.^5^ mBC is associated with poor outcomes, including a 5-year relative survival rate of 30%.^4^ In the 8 years since the approval of the first cyclin-dependent kinase 4/6 inhibitor (CDK4/6i), palbociclib, a CDK4/6i in combination with endocrine therapy (ET) has emerged as the standard of care as a first-line (1L) treatment option for adults with HR+/HER2– mBC.^6-8^ Although palbociclib was first approved for use in combination with an aromatase inhibitor (AI) for postmenopausal women with HR+/HER2− mBC,^6^ its label has since been expanded to include indications for combinatorial use with fulvestrant^9^ and the treatment of men^10^ and premenopausal women^11^ with mBC.

Phase II (PALOMA-1) and phase III (PALOMA-2) randomized clinical trials (RCTs) have demonstrated efficacy in prolonging progression-free survival (PFS) for patients treated with a combination of palbociclib and letrozole when compared with letrozole alone or letrozole plus placebo.^12,13^ In PALOMA-1, the median PFS was significantly longer with palbociclib plus letrozole (20.2 months) than with ­letrozole alone (10.2 months) (hazard ratio [HR], 0.49; 95% CI, 0.32-0.75; *P* = .0004). Similarly, PALOMA-2 reported a significantly longer median PFS with palbociclib plus letrozole (24.8 months) than with placebo plus letrozole (14.5 months) (HR, 0.58; 95% CI, 0.46-0.72; *P* < .001). Although the median overall survival (OS) was numerically longer for patients treated with palbociclib plus letrozole (37.5 months) in PALOMA-1, it was not significantly different than those treated with letrozole alone (34.5 months) (HR, 0.90; 95% CI, 0.62-1.29; *P* = .28).^14^ Data from the PALOMA-2 trial also showed a nonsignificant but numerically longer median OS for patients in the palbociclib plus letrozole group (53.9 months) relative to those in the placebo plus letrozole group (51.2 months) (HR, 0.96; 95% CI, 0.78-1.18; *P* = .34).^15^

Although data from RCTs such as the PALOMA studies are essential for determining a drug’s therapeutic value under tightly controlled conditions, certain patient demographics are frequently underrepresented. For example, the PALOMA-1 and -2 trials included very few African American participants (PALOMA-1, *n* = 2, [1.2%]; PALOMA-2, *n* = 11, [1.7%]).^13,14^ Indeed, multiple reports have highlighted underrepresentation of African American patients in clinical trials.^16,17^ Real-world evidence (RWE) studies are important complementary analyses that provide data on the effectiveness of treatments in routine clinical practice and among more diverse patient cohorts than are typically found in RCTs. Findings from multiple real-world analyses have demonstrated the effectiveness of palbociclib plus ET in extending OS and real-world progression-free survival (rwPFS) relative to AI alone.^7,18-22^

Previous RWE studies have been limited by relatively short follow-up times, small sample sizes, and the lack of ET monotherapy comparator arms. To address these shortcomings, the Palbociclib-REAl-world first-LIne comparaTive effectiveness studY eXtended (P-REALITY X)^23^ has continued to leverage the size and diversity of the Flatiron Health Analytic Database and has extended potential follow-up to 68 months and expanded patient sample size (*n* = 2888) relative to previous studies.^24,25^ Results from this study revealed significantly longer OS and rwPFS for patients treated with 1L palbociclib plus an AI versus an AI alone.^23^ Following stabilized inverse probability treatment weighting (sIPTW), the median OS was 49.1 months (95% CI, 45.2-57.7) in the palbociclib plus AI group and 43.2 months (95% CI, 37.6-48.0) in the AI-alone group (hazard ratio [HR], 0.76; 95% CI, 0.65-0.87; *P* < .0001). Following sIPTW, median rwPFS was 19.3 months (95% CI, 17.5-20.7) in the palbociclib plus AI group and 13.9 months (95% CI, 12.5-15.2) in the AI-alone group (HR, 0.70; 95% CI, 0.62-0.78; *P* < .0001).

In this retrospective study, we assessed real-world patient data from the Flatiron Health Analytic Database and report OS and rwPFS effectiveness outcomes for African American patients with HR+/HER2− mBC who received 1L treatment with either palbociclib plus an AI or an AI alone. Furthermore, we provide dose-adjustment data for African American patients receiving palbociclib.

## Methods

### Study Design and Data Source

The Flatiron Health longitudinal database contains electronic health records (EHRs)-derived data from more than 280 cancer clinics representing more than 3 million actively treated patients with cancer in the US. This retrospective analysis used deidentified EHRs from patients in the Flatiron database, and detailed methods have been previously described.^23^ Included patients were African American women or men aged ≥ 18 years with HR+/HER2− mBC who were prescribed palbociclib plus an AI or an AI alone as 1L therapy between February 2015 and March 2020. Patients were evaluated from therapy initiation until September 30, 2020 (data cutoff date), death, or last visit, whichever came first. Patients were excluded if they had previously been treated with another CDK4/6i, an AI, tamoxifen, raloxifene, toremifene, ­fulvestrant, or chemotherapy for mBC. Patients were also excluded if they were treated with a CDK4/6i as part of a clinical trial. Furthermore, patients were excluded if they received their first therapy (captured in structured EHR fields) > 90 days after their mBC diagnosis.^26^

### Outcomes

OS, defined as months from the initiation of treatment with palbociclib plus an AI or an AI alone to death, was assessed in this analysis.^25^ Death date was determined using a consensus variable across the 3 data sources (EHR, Social Security Death Index, and a commercial death dataset) that has been previously benchmarked against the National Death Index.^27^ Using this mortality variable provides good sensitivity and specificity in mortality surveillance to reduce concerns of bias in OS estimates.^27,28^ Surviving patients were censored at the study cutoff date (September 30, 2020).

rwPFS, defined as months from the start of palbociclib plus an AI or an AI alone to death or disease progression,^25,29^ was also assessed. Disease progression was determined by the treating clinician based on radiology, tissue biopsy, laboratory evidence, or clinical assessment. If patients did not die or experience disease progression, those with ≥ 2 lines of therapy (LOT) were censored at the date of initiation of the next LOT, and those with one LOT were censored at their last visit date during the study period.

### Statistical Analysis

Descriptive statistics were used for demographic and clinical characteristics. Median survival times were calculated using Kaplan–Meier survival analysis. Cox proportional hazards models without and with adjusting for baseline demographics and clinical characteristics (Table 1) were used to estimate the relative effectiveness of palbociclib plus an AI versus an AI alone. sIPTW was used to balance patient demographic and clinical characteristics as a sensitivity analysis to assess the robustness of the multivariable analyses. All analyses were performed using SAS version 9.1.4 or higher (SAS Institute, Cary, NC, USA).

**Table 1.** Patient characteristics.

Characteristics	Palbociclib + AI (*n* = 127)	AI alone (*n* = 143)	Standardized difference
Age, year			
Mean (SD)	62.2 (12.2)	68.7 (10.0)	−0.5833
Median (IQR)	64.0 (15.0)	68.0 (18.0)	
Age group,^a^ year			
18-49	17 (13.4)	2 (1.4)	0.4707
50-64	51 (40.2)	51 (35.7)	0.0927
65-74	40 (31.5)	42 (29.4)	0.0462
≥ 75	19 (15.0)	48 (33.6)	−0.4446
Sex			
Male	1 (0.8)	2 (1.4)	−0.0588
Female	126 (99.2)	141 (98.6)	
Practice type^a^			
Community	119 (93.7)	134 (93.7)	−0.0002
Academic	8 (6.3)	9 (6.3)	
Disease stage at initial diagnosis^a^			
I	10 (7.9)	16 (11.2)	−0.1131
II	32 (25.2)	37 (25.9)	−0.0155
III	16 (12.6)	36 (25.2)	−0.3255
IV	62 (48.8)	44 (30.8)	0.3752
Not documented	7 (5.5)	10 (7.0)	−0.0612
ECOG performance status^a^			
0	52 (40.9)	32 (22.4)	0.4074
1	26 (20.5)	33 (23.1)	−0.0631
2, 3, or 4	17 (13.4)	28 (19.6)	−0.1675
Not documented	32 (25.2)	50 (35.0)	−0.2142
Visceral disease^a^^,^^b^			
No	92 (72.4)	101 (70.6)	0.0402
Yes	35 (27.6)	42 (29.4)	
Bone-only disease^a^^,^^c^			
No	77 (60.6)	94 (65.7)	−0.1060
Yes	50 (39.4)	49 (34.3)	
Brain metastases			
No	125 (98.4)	135 (94.4)	0.2175
Yes	2 (1.6)	8 (5.6)	
Interval from initial BC diagnosis to mBC,^a^ year			
De novo mBC	62 (48.8)	44 (30.8)	0.3752
0-1	5 (3.9)	5 (3.5)	0.0233
1-5	25 (19.7)	54 (37.8)	−0.4077
> 5	35 (27.6)	40 (28.0)	−0.0092
Number of metastatic sites^a^^,^^d^			
1	69 (54.3)	76 (53.2)	0.0237
2	35 (27.6)	24 (16.8)	0.2616
3	9 (7.1)	10 (7.0)	0.0037
4	9 (7.1)	3 (2.1)	0.2400
≥ 5	0 (0.0)	5 (3.5)	−0.2692
Not documented	5 (3.9)	25 (17.5)	−0.4489
Median follow-up duration (IQR), month	24.0 (14.5-36.3)	18.2 (9.9-35.4)	

Data presented as *n* (%), unless specified otherwise.

^a^Covariates used in multivariable Cox proportional hazards models.

^b^Visceral disease is defined as metastatic disease in the lung and/or liver; patients could have had other sites of metastases.

^c^Bone-only disease is defined as metastatic disease in the bone only.

^d^Multiple metastases at the same site were counted as one site (eg, 3 bone metastases in the spine was considered only one site).

Abbreviations: AI: aromatase inhibitor; BC: breast cancer; ECOG: Eastern Cooperative Oncology Group; IQR: interquartile range; mBC: metastatic breast cancer.

## Results

### Patients

A total of 270 African American patients from the Flatiron database initiated treatment with palbociclib plus an AI (*n* = 127, 47.0%) or with an AI alone (*n* = 143, 53.0%) as 1L therapy from February 3, 2015 to March 31, 2020 (Table 1). Follow-up time was up to 68 months from the index date to the study cutoff date of September 30, 2020. Median follow-up was 24.0 months for patients who received palbociclib plus an AI and 18.2 months for patients who received an AI alone. Median age was 64.0 years in patients treated with palbociclib plus an AI and 68.0 years in patients treated with an AI alone. Compared with patients treated with an AI alone, those treated with palbociclib plus an AI were more likely to have de novo mBC (48.8% vs. 30.8%). Patient characteristics after sIPTW are shown in Supplementary Table. Most patient characteristics were generally balanced except for disease stage at initial diagnosis, interval from initial BC diagnosis to mBC, brain metastasis, and number of metastatic sites.

### Palbociclib Dose Adjustment

**Figure 1.**
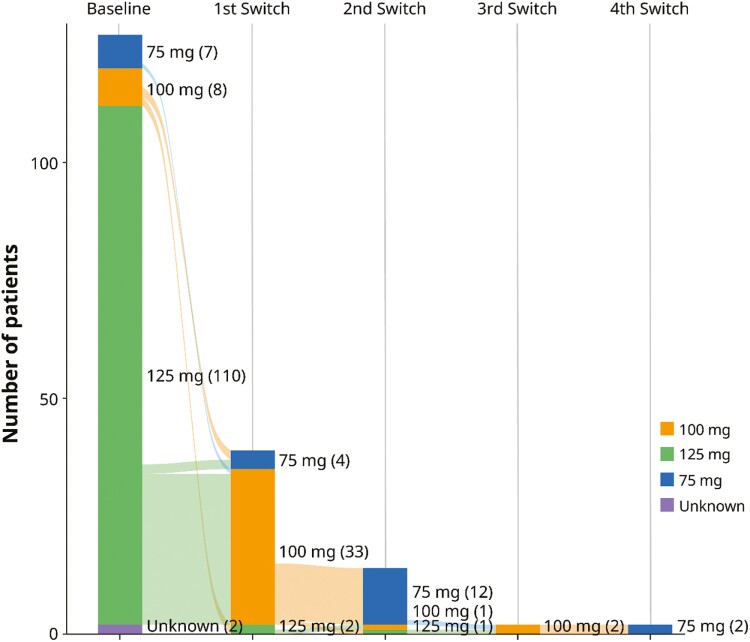
Sankey diagram indicating palbociclib dose adjustments from initial doses during a median follow-up of 24 months in African American patients.

Of the patients treated with palbociclib (*n* = 127), 110 (86.6%) started at 125 mg/day, 8 (6.3%) at 100 mg/day, and 7 (5.5%) at 75 mg/day. Dose adjustment, increase, or decrease was experienced by 30.7% of all patients receiving palbociclib. For patients receiving an initial dose of 125 mg/day, 30.9% (34/110) had dose adjustments compared with 50% (4/8) of those who started at 100 mg/day and 14.3% (1/7) of those who started at 75 mg/day. Among the patients who started at 125 mg/day and had their dose reduced (*n* = 34), 32 had their dose reduced to 100 mg/day and 2 had their dose reduced to 75 mg/day. Of the 32 patients who had a dose reduction to 100 mg/day, 11 had a subsequent single reduction to 75 mg/day, and 2 patients experienced 4 dose changes including a final reduction to 75 mg/day. Among patients with a starting dose of 100 mg/day who received dose adjustments (*n* = 4), 2 had their dose decreased to 75 mg/day, and 2 had their dose increased to 125 mg/day. One of the patients who had a dose increase from 100 to 125 mg/day was subsequently returned to 100 mg/day. Only one patient with an initial dose of 75 mg/day had a dose adjustment, which was an increase to 100 mg/day. Among patients with dose adjustments, the median time to first dose adjustment was 91.5 days among patients initiating palbociclib at 125 mg/day (*n* = 34), 213 days among those who started at 100 mg/day (*n* = 4), and 168 days among those who started at 75 mg/day (*n* = 1). A Sankey diagram summarizes the dose adjustments in Fig. 1.

### Overall Survival

**Figure 2.**
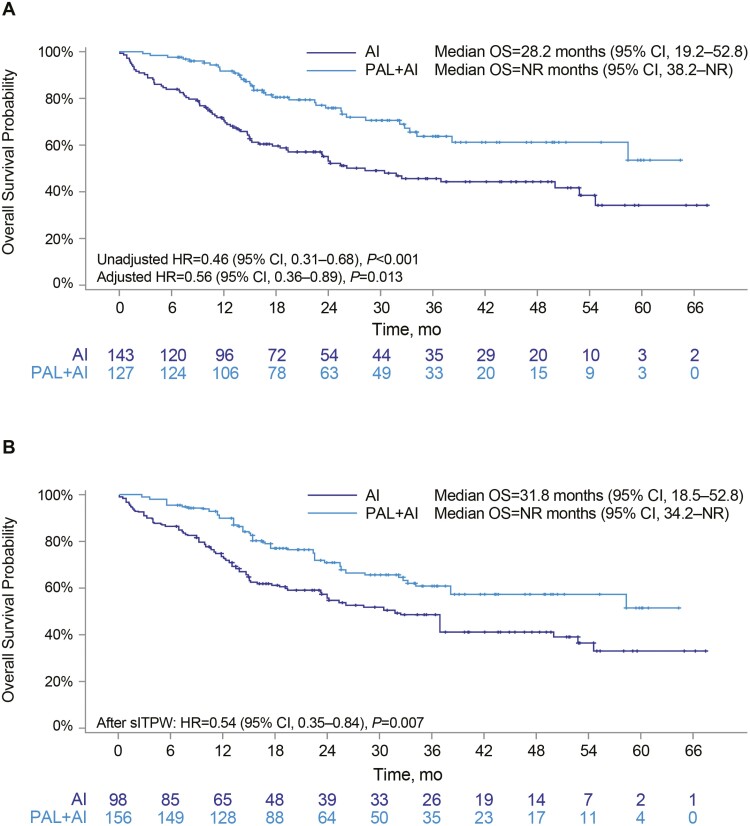
Kaplan–Meier curves of OS in African American patients: Unadjusted (**A**) and after sIPTW (**B**). Abbreviations: AI: aromatase inhibitor; CI: confidence interval; HR: hazard ratio; mo: month; NR: not reached; OS: overall survival; PAL: palbociclib; sIPTW: stabilized inverse probability of treatment weighting.

In the unadjusted analysis (*n* = 270), median OS was significantly longer among patients treated with palbociclib plus an AI versus patients treated with an AI alone (not reached [NR], 95% CI, 38.2-NR, vs. 28.2 months, 95% CI, 19.2-52.8; HR, 0.46; 95% CI, 0.31-0.68; *P* < .001; Fig. 2A; Table 2). The results were similar following adjustment based on multivariable Cox regression analysis (HR, 0.56; 95% CI, 0.36-0.89; *P* = .013) and sIPTW sensitivity analysis (HR, 0.54; 95% CI, 0.35-0.84; *P* = .007; Fig. 2A, 2B). OS rates were higher for patients treated with palbociclib plus an AI than for those treated with an AI alone at 12 months (91.7% vs. 70.3%, respectively), 24 months (75.9% vs. 53.2%), and 36 months (61.2% vs. 44.3%) (Table 2).

**Table 2.** OS and rwPFS in African American patients.

Outcome	Palbociclib + AI (*n *= 127)	AI Alone (*n* = 143)
OS rate, %		
At 12 months	91.7	70.3
At 24 months	75.9	53.2
At 36 months	61.2	44.3
Median OS, months (95% CI)	NR (38.2-NR)	28.2 (19.2-52.8)
HR (unadjusted), (95% CI)	0.46 (0.31-0.68), *P* < .001	
HR (multivariable adjusted), (95% CI)	0.56 (0.36-0.89), *P* = .013	
HR (after sIPTW), (95% CI)	0.54 (0.35-0.84), *P = *.007	
rwPFS rate, %		
At 6 months	78.4	66.9
At 12 months	59.9	43.9
At 20 months	46.6	30.1
Median rwPFS, months (95% CI)	18.0 (12.4-26.7)	10.5 (7.0-13.4)
HR (unadjusted), (95% CI)	0.63 (0.44-0.88), *P* = .007	
HR (multivariable adjusted), (95% CI)	0.74 (0.47-1.17), *P* = .199	
HR (after sIPTW), (95% CI)	0.72 (0.48-1.07), *P* = .102	

Abbreviations: AI: aromatase inhibitor; CI: confidence interval; HR: hazard ratio; NR: not reached; OS: overall survival; rwPFS: real-world progression-free survival; sIPTW: stabilized inverse probability of treatment weighting.

### Real-World Progression-Free Survival

**Figure 3.**
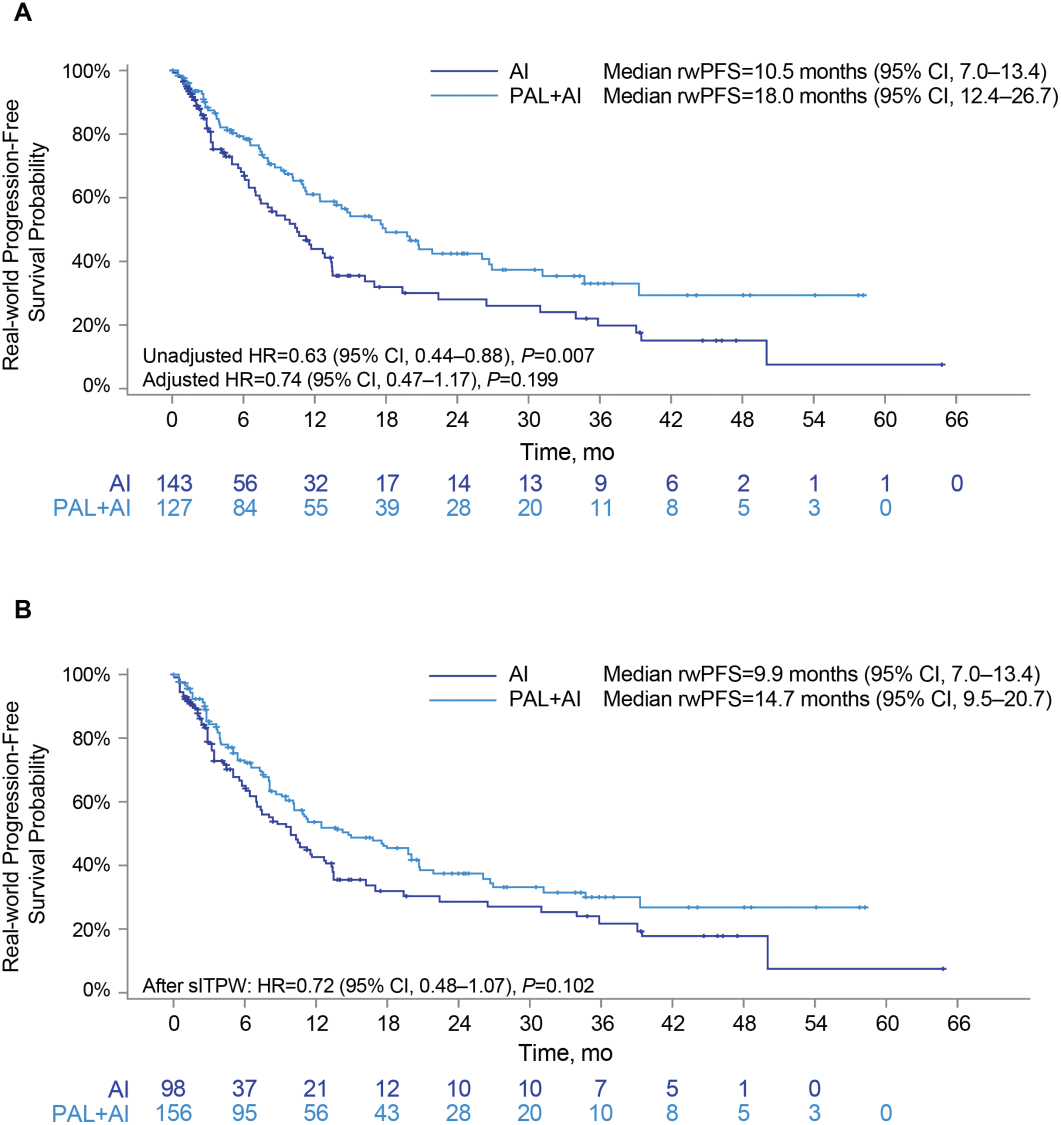
Kaplan–Meier curves of rwPFS in African American patients: Unadjusted (**A**) and after sIPTW (**B**). Abbreviations: AI: aromatase inhibitor; CI: confidence interval; HR: hazard ratio; mo: month; NR: not reached; PAL: palbociclib; rwPFS: real-world progression-free survival; sIPTW: stabilized inverse probability of treatment weighting.

Median rwPFS was significantly longer for patients treated with palbociclib plus an AI than those treated with an AI alone (18.0 months, 95% CI, 12.4-26.7 vs. 10.5 months, 95% CI, 7.0-13.4; unadjusted HR, 0.63; 95% CI, 0.44-0.88; *P* = .007; Fig. 3A; Table 2). However, the difference between treatment arms was not significant following multivariable adjustment (HR, 0.74; 95% CI, 0.47-1.17; *P* = .199) or sIPTW sensitivity analysis (HR, 0.72; 95% CI, 0.48-1.07; *P* = .102; Fig. 3A, 3B). rwPFS rates were higher for the palbociclib plus AI group than the AI-alone group at 6 months (78.4% vs. 66.9%), 12 months (59.9% vs. 43.9%), and 20 months (46.6% vs. 30.1%) (Table 2).

## Discussion

Despite recent progress in ameliorating racial disparities in cancer outcomes, there remains a compelling need to close gaps in BC mortality between African American and other patient populations. Although RCTs are the gold standard for determining efficacy of treatments, they can feature highly restrictive inclusion criteria that can result in the underrepresentation of certain patient subpopulations, including African Americans.^30^ However, real-world studies, drawing on large databases that include diverse patients, can provide data on the effectiveness of therapies for African American patients in routine clinical settings. In this real-world analysis of 270 African American patients with HR+/HER2− mBC, we found that palbociclib plus an AI was more effective than an AI alone in extending OS and rwPFS.

The extended median OS in African Americans treated with palbociclib plus AI compared with AI alone observed in our study (*n* = 270, adjusted HR = 0.56; 95% CI, 0.36-0.89) is similar to what was observed in other studies that have used the Flatiron database. These Flatiron-based analyses reported prolonged OS in African American subgroups: women with HR+/HER2− mBC treated with palbociclib plus letrozole versus letrozole alone (*n* = 118, HR, 0.45; 95% CI, 0.25-0.79)^25^ and postmenopausal women and men with HR+/HER2− mBC treated with palbociclib plus an AI versus an AI alone (P-REALITY X; *n* = 230, HR, 0.44; 95% CI, 0.27-0.70).^23^ However, data on OS outcomes for African American patients treated with palbociclib from other EHR databases are scarce. The multinational Ibrance Real World Insights (IRIS) study, which retrospectively reviewed medical records of women with HR+/HER2− mBC or advanced BC, reported 24-month survival rates of 93.2% (95% CI, 83.8-97.2) for non-Hispanic Black patients (*n* = 96) treated with palbociclib plus an AI, which was similar to the survival rate of the overall population (*n* = 1732; 90.3%; 95% CI, 86.8-92.9).^31^ This finding compares favorably with the 75.9% 24-month survival rate in our study, although the lack of an AI alone comparator arm in the IRIS study precludes direct comparison. Interestingly, another Flatiron-based study reported that non-Hispanic Black women with HR+/HER2− mBC had poorer median OS than White women after second-line initiation of CDK4/6is, most likely due to outcomes of non-Hispanic Black women who did not receive a CDK4/6i during 1L treatment.^32^

The prolonged OS observed in the Flatiron database real-world studies with palbociclib treatment was also observed in the PALOMA-1 and -2 trials, although only numerically longer.^13,14^ While results of RCTs and RWE studies are not directly comparable, they are complementary tools that can be used to understand the treatment landscape. A variety of reasons could account for the disparities between the PALOMA trials and the Flatiron-based studies, such as differences in sample size, follow-up length, primary endpoints, patient characteristics, and healthcare settings.^23^ The larger sample size and longer follow-up times in these RWE studies relative to the PALOMA trials may be more apt at detecting significant changes in OS with 1L palbociclib plus AI combination therapy, especially for underrepresented subgroups such as African American patients.

While our results showing longer OS in the palbociclib plus AI group versus the AI alone group were robust, the HR for rwPFS was no longer significant following multivariable adjustment or sIPTW sensitivity analysis. Nevertheless, the rwPFS adjusted HRs of 0.72-0.74 that we report here are clinically meaningful and support the effectiveness of palbociclib plus an AI versus an AI alone. The other Flatiron-based studies also showed prolonged rwPFS in African Americans with palbociclib treatment.^23,25^ Furthermore, the IRIS study reported a 12-month progression-free rate of 81.8% in the non-Hispanic Black subgroup that compares favorably with the 59.9% rate observed in this study, although the lack of an AI alone arm in the IRIS study prevents direct comparisons.^31^ Finally, both PALOMA-1 and -2 trials reported significantly prolonged PFS with palbociclib with an AI than an AI alone in the overall populations.^12,13^ Overall, our findings add to a preponderance of evidence from both RCTs and real-world studies that supports the benefit of palbociclib plus AI for extending PFS in both African American patients and the overall patient population.^12,13,23-25^

Important additional outcomes that were assessed in this study are initial palbociclib dose and patterns of dose adjustment. In line with previous real-world studies, 11.8% of patients initiated palbociclib at less than the label-recommended 125 mg/day dose.^11,19,31^ While previous findings have shown that OS and PFS are shorter when ­palbociclib doses are < 125 mg/day,^19^ label indications recommend starting patients on < 125 mg/day if they have severe hepatic impairment or are concurrently taking a strong CYP3A inhibitor.^11^ In addition, 30.1% of patients with a starting dose of 125 mg/day experienced dose adjustment, similar to results from a previous RWE study (39.2%)^19^ and the PALOMA-1 and PALOMA-2 trials (39.8% and 39.4%, respectively).^12,33^ Clinicians’ reasoning for reduced initial dosing and for dose changes is not available in the Flatiron database and should be investigated in future studies.

This study has several important strengths. Previous research on the effectiveness of therapies for African American patients with mBC in both clinical trials and real-world studies is limited, and this study provides valuable evidence for the use of palbociclib plus an AI in this patient population in real-world clinical practice. The size and diversity of the Flatiron database allow for analysis of patients, such as African Americans who have been underrepresented in clinical trials, and fills a gap in the medical literature that serves to aid in clinical decision-making. In addition, the date of death was validated in the Flatiron Health database and follow-up time was up to 68 months.

However, this study also has some limitations. This is a retrospective database analysis, and therefore causal relationships between palbociclib treatment and patient outcomes cannot be drawn. Incomplete or missing data documented in the EHRs, such as ECOG performance status and comorbidities, may have led to bias. In addition, there were a few notable differences between treatment groups at baseline (eg, age, disease stage at initial diagnosis, interval from initial BC diagnosis to mBC, brain metastasis, and number of metastatic sites). Although multivariable analysis and sIPTW allowed us to statistically control for these differences when comparing clinical outcomes between palbociclib plus an AI versus an AI alone, these differences may reflect real-world clinicians’ treatment decision-making on the basis of patient characteristics, which are associated with prognosis.^34^ In addition, disease progression was not assessed on a schedule as in clinical trials and was not based on Response Evaluation Criteria in Solid Tumors (RECIST). As a result, the data are limited by the individual treating clinician’s interpretation of radiographic scans or pathology results. Although multivariable analyses and sIPTW were conducted, the effects of unobserved confounders on the results presented here could not be excluded. Although the sample size of 270 African American patients is relatively large compared with other studies of this population, statistical power may still be limited. Finally, this analysis may not be generalizable to patients outside of the Flatiron network.

African American patients with mBC continue to have higher mortality than White patients. Known factors underlying these outcomes include later stage diagnosis^35^ and reduced access to quality health care.^36^ However, even in clinical trials with stratified randomization and consistent quality of care among patients, African American patients still experience worse outcomes, indicating that additional factors underlie observed racial disparities.^37^ Emerging evidence from research examining the intrinsic molecular subtypes within HR+/HER2− BC suggests that young African American women may be more likely than their White peers to present with nonluminal A HR+ subtypes that are associated with poor outcomes.^38^ Furthermore, a recent study evaluating the efficacy of palbociclib by intrinsic molecular subtype in HR+/HER2− tumors noted better PFS for patients with luminal subtypes than for patients with nonluminal subtypes.^39^ These findings highlight the need for future studies that recognize the heterogeneity of HR+/HER2− molecular subtypes while identifying reasons for persistent racial disparities in mBC patient outcomes.

## Conclusion

Overall, this comparative analysis of palbociclib plus an AI versus an AI alone indicates that 1L palbociclib in combination with AI is associated with improved effectiveness for treatment of African American patients with HR+/HER2− mBC in real-world settings. Together with future mBC RCTs that are more inclusive of African American patients, complementary findings from larger RWE and safety studies are warranted.

## Supplementary Material

oyad209_suppl_Supplementary_Material

## Data Availability

The deidentified data that support this study’s findings from Flatiron Health, Inc. are available upon request subject to a license agreement. Please contact DataAccess@flatiron.com to determine licensing terms and for access to the training, data dictionary, validation, and data sets, or The Flatiron Health Analytic Database at https://flatiron.com/contact/.
