# Supplementary material for: Real-World Effectiveness of Palbociclib Plus Aromatase Inhibitors in African American Patients With Metastatic Breast Cancer
Source: Oncologist. 2023 Jul 24;28(10):866–74. doi: 10.1093/oncolo/oyad209 (PMC10546832; doi:10.1093/oncolo/oyad209)
Supplement: oyad209_suppl_Supplementary_Material [file oyad209_suppl_supplementary_material.docx]

**Supplementary Material**

**Supplementary Table.** Patient characteristics after sIPTW

| **Characteristic** | **Palbociclib + AI**  **(*n* = 156)** | **AI Alone**  **(*n* = 98)** | **Standardized Difference** |
| --- | --- | --- | --- |
| Age, y |  |  |  |
| Mean (SD) | 65.5 (13.4) | 66.3 (9.3) | -0.0652 |
| Median (IQR) | 65.0 (18.0) | 66.0 (16.0) |  |
| Age group, y |  |  |  |
| 18-49 | 11 (7.2) | 6 (6.2) | 0.0402 |
| 50-64 | 61 (38.7) | 38 (38.7) | 0.0002 |
| 65-74 | 44 (27.9) | 29 (29.7) | -0.0402 |
| ≥ 75 | 41 (26.2) | 25 (25.4) | 0.0185 |
| Sex |  |  |  |
| Male | 2 (1.0) | 2 (1.6) | -0.0549 |
| Female | 155 (99.0) | 97 (98.4) |  |
| Practice type |  |  |  |
| Community | 145 (92.7) | 88 (90.0) | 0.0929 |
| Academic | 11 (7.4) | 10 (10.0) |  |
| Disease stage at initial diagnosis |  |  |  |
| I | 14 (9.3) | 9 (9.5) | -0.0090 |
| II | 37 (23.9) | 23 (23.4) | 0.0107 |
| III | 25 (16.0) | 21 (21.7) | -0.1482 |
| IV | 63 (40.6) | 38 (38.5) | 0.0426 |
| Not documented | 16 (10.3) | 7 (6.8) | 0.1255 |
| ECOG performance status |  |  |  |
| 0 | 49 (31.4) | 28 (28.5) | 0.0647 |
| 1 | 35 (22.3) | 24 (24.6) | -0.0543 |
| 2, 3, or 4 | 31 (19.5) | 16 (16.7) | 0.0735 |
| Not documented | 42 (26.8) | 30 (30.3) | -0.0775 |
| Visceral disease ^a^ |  |  |  |
| No | 116 (74.1) | 74 (75.2) | -0.0254 |
| Yes | 41 (26.0) | 24 (24.8) |  |
| Bone-only disease ^b^ |  |  |  |
| No | 96 (61.7) | 63 (63.7) | -0.0414 |
| Yes | 60 (38.3) | 36 (36.3) |  |
| Brain metastases |  |  |  |
| No | 155 (98.9) | 89 (90.2) | 0.3909 |
| Yes | 2 (1.1) | 10 (9.8) |  |
| Interval from initial BC diagnosis to mBC, y |  |  |  |
| De novo mBC | 63 (40.6) | 38 (38.5) | 0.0426 |
| 0-1 | 8 (4.8) | 3 (3.1) | 0.0889 |
| 1-5 | 34 (21.9) | 34 (34.8) | -0.2891 |
| > 5 | 51 (32.7) | 23 (23.6) | 0.2029 |
| Number of metastatic sites^c^ |  |  |  |
| 1 | 87 (55.7) | 56 (56.8) | -0.0220 |
| 2 | 33 (21.1) | 21 (20.9) | 0.0049 |
| 3 | 9 (5.7) | 7 (7.1) | -0.0554 |
| 4 | 10 (6.2) | 2 (1.6) | 0.2405 |
| ≥ 5 | 0 (0.0) | 3 (2.7) | -0.2349 |
| Not documented | 18 (11.4) | 11 (11.0) | 0.0106 |
| Median follow-up duration (IQR), mo | 18.9 (13.8–34.0) | 17.2 (9.2–36.9) |  |

Data presented as n (%), unless specified otherwise. ^a^Visceral disease is defined as metastatic disease in the lung and/ or liver; patients could have had other sites of metastases; ^b^Bone-only disease is defined as metastatic disease in the bone only; ^c^Multiple metastases at the same site were counted as 1 site (e.g., 3 bone metastases in the spine was considered only 1 site). AI, aromatase inhibitor; BC, breast cancer; ECOG, Eastern Cooperative Oncology Group; IQR, interquartile range; mBC, metastatic breast cancer; SD, standard deviation; sIPTW, stabilized inverse probability of treatment weighting; y, years.
